# PFAS concentrations in early and mid-pregnancy and risk of gestational diabetes mellitus in a nested case-control study within the ethnically and racially diverse PETALS cohort

**DOI:** 10.1186/s12884-023-05953-3

**Published:** 2023-09-13

**Authors:** Alicia K. Peterson, Yeyi Zhu, Sophia Fuller, Juanran Feng, Stacey Alexeeff, Susanna D. Mitro, Kurunthachalam Kannan, Morgan Robinson, Amy Padula, Assiamira Ferrara

**Affiliations:** 1 Division of Research, Kaiser Permanente Northern California, 2000 Broadway, Oakland, CA 94612 USA; 2 Center for Upstream Prevention of Adiposity and Diabetes Mellitus (UPSTREAM), Division of Research, Kaiser Permanente Northern California, 2000 Broadway, Oakland, CA 94612 USA; 3 Department of Pediatrics, New York University School of Medicine, 145 East 32 Street, New York, NY 10016 USA; 4 Department of Obstetrics, Gynecology and Reproductive Sciences, University of California San Francisco, 490 Illinois Street, San Francisco, 94143 CA USA

**Keywords:** PFAS, GDM, Gestational diabetes, BKMR, Pregnancy

## Abstract

**Background:**

Per- and polyfluoroalkyl substances (PFAS) are persistent synthetic chemicals and are commonly found in everyday items. PFAS have been linked to disrupting glucose homeostasis, however, whether they are associated with gestational diabetes mellitus (GDM) risk remains inconclusive. We examined prospective associations of PFAS concentrations measured twice in pregnancy with GDM risk.

**Methods:**

In the PETALS pregnancy cohort, a nested case–control study which included 41 GDM cases and 87 controls was conducted. PFAS analytes were measured in blood serum collected in both early and mid-pregnancy (mean [SD]: 13.9 [2.2] and 20.2 [2.2] gestational weeks, respectively), with cumulative exposure calculated by the area-under-the-curve (AUC) to integrate both the PFAS concentration and the timing of the exposure. Individual adjusted weighted unconditional logistic regression models examined seven PFAS in association with GDM risk. P-values were corrected using the false-discovery-rate (FDR). Mixture models were analyzed with Bayesian kernel machine regression (BKMR).

**Results:**

PFDA, PFNA and PFOA were individually associated with higher GDM risk per interquartile range (IQR) in early pregnancy (OR [95% CI]: 1.23 [1.09, 1.38]), 1.40 [1.24, 1.58]), and 1.15 [1.04, 1.27], respectively), mid-pregnancy (1.28 [1.15, 1.43], 1.16 [1.05, 1.28], and 1.20 [1.09, 1.33], respectively), and with cumulative exposure (1.23 [1.09, 1.38], 1.21 [1.07, 1.37], and 1.19 [1.09, 1.31], respectively). PFOS in mid-pregnancy and with cumulative exposure was associated with increased GDM risk (1.41 [1.17, 1.71] and 1.33 [1.06, 1.58], respectively). PFUnDA in early pregnancy was associated with lower GDM risk (0.79 [0.64, 0.98]), whereas mid-pregnancy levels were associated with higher risk (1.49 [1.18, 1.89]). PFHxS was associated with decreased GDM risk in early and mid-pregnancy (0.48 [0.38, 0.60] and 0.48 [0.37, 0.63], respectively) and with cumulative exposure (0.49 [0.38,0.63]). PFPeA was not associated with GDM. Similar conclusions were observed in BKMR models; however, overall associations in these models were not statistically significant.

**Conclusions:**

Higher risk of GDM was consistently observed in association with PFDA, PFNA, and PFOA exposure in both early and mid-pregnancy. Results should be corroborated in larger population-based cohorts and individuals of reproductive age should potentially avoid known sources of PFAS.

**Supplementary Information:**

The online version contains supplementary material available at 10.1186/s12884-023-05953-3.

## Background

Gestational diabetes mellitus (GDM) is a common complication of pregnancy (7.8 per 100 US births in 2020), with sharp increases in incidence rates observed over recent years [1–3]. These rising rates are a public health concern given that GDM is associated with placental changes in pregnancy [4, 5], requires prompt and extensive management during pregnancy [6], and is associated with adverse perinatal outcomes and long-term health outcomes for both the mother and child [7]. Specifically, GDM is a predictor of maternal type 2 diabetes post-pregnancy [8, 9] and delivering an infant large for gestational age with possible adverse cardiometabolic phenotypes, including obesity, metabolic syndrome and type 2 diabetes [10–12]. Differences in prevalence rates have been documented across racial/ethnic groups with the highest rates in Asian, followed by Hispanic and Black, and lowest in non-Hispanic White [2, 3]. Established risk factors for GDM include advanced age at pregnancy, pre-pregnancy obesity, and excessive gestational weight gain [13–16]. In addition, recent literature has suggested that environmental factors, particularly exposure to endocrine-disrupting chemicals (EDCs) including per-and polyfluoroalkyl substances (PFAS) [17], may additionally play an important role in the risk of GDM [18].

PFAS are a large class of persistent synthetic chemicals used in numerous industrial and consumer products over recent decades due to their water and oil resistant properties which has resulted in widespread infiltration into the environment [19–22]. A common exposure route of PFAS to humans is through ingestion from the diet as compounds have been commonly found in drinking water and fish or being transferred into food from fast-food packaging and non-stick (i.e., Teflon) pans [23–27]. Pregnancy may be a susceptible exposure period with heightened sensitivity to these compounds due to biological alterations occurring during gestation that are regulated by the endocrine system with EDCs having the possibility to affect various physiological processes [28].

PFAS exposure during gestation has been associated with miscarriage, low birthweight, reduced fetal growth, preterm birth, and preeclampsia [29–33], and concentration levels have shown to differ based on race and ethnicity [34–36]. Most studies assessing the influence of prenatal PFAS exposure on GDM risk have been conducted in Chinese populations with varying results, although positive associations have been suggested [37–43]. Studies conducted in European and North American populations of predominantly non-Hispanic White participants have indicated PFAS exposure to be associated with higher blood glucose levels in pregnancy, but results remain largely inconsistent [44–48]. In addition, previous studies have primarily only measured PFAS concentrations at a single timepoint in pregnancy, limiting the ability to determine how exposure across pregnancy influences risk, as PFAS levels have shown to fluctuate across pregnancy [49].

This case–control study, nested within the diverse prospective Pregnancy Environment and Lifestyle Study (PETALS) cohort of pregnant individuals, assesses the associations of seven PFAS [perfluorooctanoic acid (PFOA), perfluorooctanesulfonic acid (PFOS), perfluorohexanesulfonic acid (PFHxS), perfluorodecanoic acid (PFDA), perfluorononanoic acid (PFNA), perfluoroundecanoic acid (PFUnDA), and perfluoro-n-pentanoic acid (PFPeA)] measured at two time points during pregnancy with the risk of GDM. We hypothesize that participants with higher levels of PFAS concentrations during gestation will have greater risk of GDM.

## Methods

### Study population

This nested case–control study included participants in PETALS, a racially and ethnically diverse population-based prospective pregnancy cohort with participants recruited between 2014 and 2017. Details related to the study design have previously been described [50]. Participants are members of Kaiser Permanente Northern California (KPNC), an integrated health care delivery system with over 4.5 million patients served and is demographically representative of the geographical coverage area [51, 52].

**Fig. 1:**
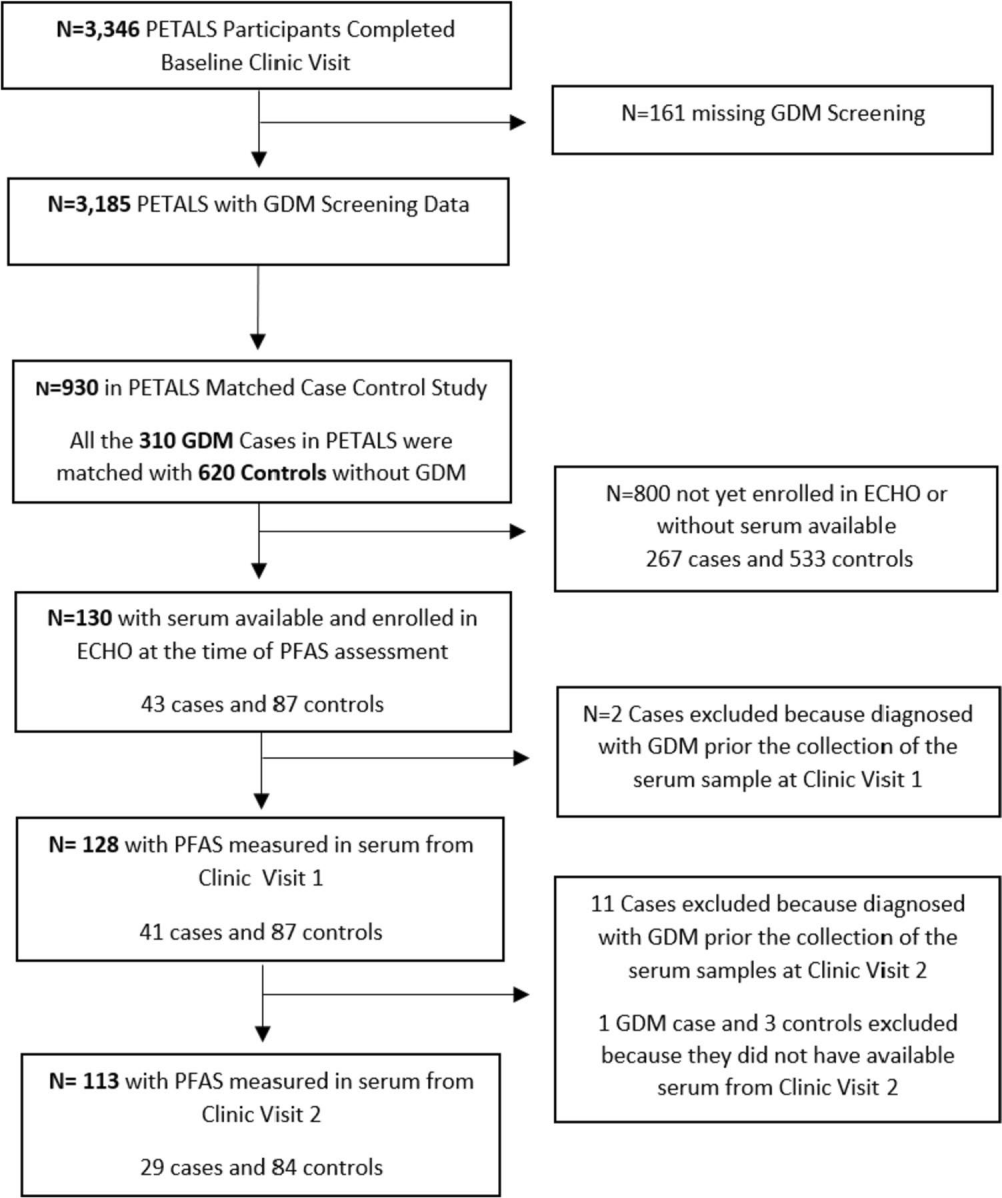
Flow Chart of Included Participants from the PETALS Cohort

The flow chart of included participants from the PETALS cohort in this current study is shown in Fig. 1. There were 3,346 pregnant individuals who were enrolled in the PETALS cohort by completing the baseline assessment (Clinic Visit 1). Among these individuals, 4.8% (*N* = 161) were missing data on GDM screening due to either a pregnancy loss (1.0%), no longer a KPNC member (1.4%), or were not screened (2.4%). Of the remaining 3,185 who were screened for GDM, there were 310 GDM cases. For the original PETALS GDM case–control study, GDM cases were matched 1:2 with non-GDM controls on age (± 5 years), calendar time for enrollment (± 3 months), gestational week at the first clinic visit (± 3 weeks), and medical facility. For this current analysis, of the 310 GDM cases and 620 matched controls there were 43 GDM cases and 87 controls with serum collected at Clinic Visit 1 (CV1) available and who were also enrolled in the National Institute of Health’s Environmental influences on Child Health Outcomes (ECHO) program [53] at the time of the PFAS measurements sponsored by ECHO. For the analysis related to PFAS assessed at CV1, we further excluded 2 GDM cases because they were diagnosed with GDM prior to the serum collection. For the analysis related to PFAS at Clinic Visit 2 (CV2), 11 GDM cases were excluded because GDM was diagnosed prior to the serum collection at CV2. In addition, 1 GDM case and 3 controls were excluded because they did not have available serum at CV2. All participants with samples at CV2 also had samples at CV1. In the current study, participants completed CV1 on average at 13.8 weeks of gestation (SD: 2.2; Range: 10.1 –19.0 gestational weeks) and is referred to in this study as early pregnancy. Participants completed CV2 on average at 20.2 weeks of gestation (SD: 2.2; Range 15.6 – 26.0 gestational weeks) and is referred to in this study as mid-pregnancy. The average time between CV1 and CV2 for participants in this study was 6.5 (SD: 0.95) weeks.

All participants in this analysis were enrolled into PETALS between 2014–2017 and provided written informed consent at time of enrollment. The human subjects committee of the Kaiser Foundation Research Institute approved all study design components. All participants included in the current analysis met the following criteria: 1) singleton gestation 2) no evidence based on medical record review of pre-existing cancer, diabetes mellitus, or liver disease (due to PFAS being associated with liver injury [54]); 3) data on GDM screening; and 4) data on PFAS concentrations measured in blood serum.

### GDM ascertainment

GDM was ascertained using the following standardized criteria implemented across KPNC: a) ≥ 2 plasma glucose values during the 100-g, 3-h oral glucose tolerance test (OGTT) meeting or exceeding the Carpenter-Coustan thresholds (≥ 5.3 mmol/L for fasting, ≥ 10.0 mmol/L for 1-h, ≥ 8.6 mmol/L for 2 h and ≥ 7.8 mmol/L for 3 h) recommended by the American College of Obstetricians and Gynecologists [55]; or b) fasting glucose ≥ 5.1 mmol/L performed alone or during the OGTT as recommended by the International Association of Diabetes and Pregnancy Study Groups and American Diabetes Association [56].

### PFAS concentrations during gestation

A fasting blood sample was collected at baseline from participants at CV1 and then again at CV2 which were conducted in early and mid-pregnancy. Serum samples were analyzed in 2020 at the Wadsworth Center's Human Health Exposure Assessment Resource (WC-HHEAR) laboratory at the NYU Langone Medical Center (Dr. Kannan's laboratory). Fourteen PFAS, specifically, PFHxS, PFOS, PFOA, PFNA, PFDA, PFUnDA, PFPeA, perfluorobutanesulfonic acid (PFBS), perfluoroheptanoic acid (PFHPA), perfluorododecanoic (PFDODA), perfluorooctanesulfonamide (PFOSA), n-ethyl perfluorooctane sulfonamido acetic acid (NETFOSAA), n-methyl perfluorooctane sulfonamido acetic acid (NMFOSAA), and perfluorohexanoic acid (PFHxA) were analyzed. The full method for the analysis of 14 PFAS in serum has previously been described [57] and further explained in the Supplemental methods. This study included analytes with at least 50% of samples above the limit of detection (LOD), as done previously [36, 58]. Values below the LOD were imputed with the LOD/$\surd$ 2 [59].

### Covariates

Covariates were identified a priori from existing literature and were visualized using a directed acyclic graph [60] (DAG) and is shown in Supplemental Fig. 1. Age, race/ethnicity, and parity information were self-reported using standardized questionnaires. Pre-pregnancy BMI was computed using weight (kg) at an average of 14 weeks prior to conception with 99% abstracted from the electronic health records and 1% being self-reported. Height (m) was measured at the first study clinic visit. Race and ethnicity was used as a covariate for a proxy of experiencing racism and discrimination and previous literature has indicated race and ethnicity is associated with both measured PFAS concentration levels [34–36], and GDM incidence rates [2, 3].

### Statistical analysis

Differences in demographic characteristics by GDM status and PFAS concentrations were determined by Wilcoxon signed rank tests, student’s t tests, Kruskal Wallis tests and Pearson’s chi square tests. Spearman correlations examined relationships across PFAS compounds at each time point and across timepoints within the same individual.

To approximate cumulative exposure across the two blood samples, the area under the curve (AUC) was calculated to integrate both the PFAS concentration and the timing of exposure assessment [61] (Equation Supplemental Fig. 2). To properly analyze the potentially broken matched trios from the original matched case–control study within PETALS, we fit adjusted weighted unconditional logistic regression models to determine the associations of individual PFAS concentrations during gestation with GDM risk separately using levels measured in early pregnancy, mid-pregnancy, and with the AUC across the two time points. Each participant was assigned an inverse of probability weight (IPW) to be used in models. IPW for cases were calculated as the inverse of the number of cases at each time point divided by the total number of cases in the PETALS cohort (*n* = 310). Controls were assigned a weight based on the inverse of the predicted probability in a logistic regression model using all PETALS controls that had GDM screening data (*n* = 2,875) with the original matching criteria. Due to the skewed PFAS distributions and for being a common approach in the literature, compounds were transformed with the natural log. PFAS analytes were then scaled by the relevant interquartile range (IQR) based on the log-transformed concentration levels for interpretations.

Since exposure to PFAS analytes occur concurrently, we assessed the effects of the seven PFAS as a mixture by fitting adjusted probit Bayesian kernel machine regression (BKMR) models on GDM risk [62]. BKMR models were fit for PFAS levels in early pregnancy, mid-pregnancy, and across both timepoints with the AUC. For the BKMR models to converge, we used a pre-adjustment method for covariates that first fit a frequentist probit regression model with no PFAS analytes but all covariates, and then incorporated the fitted values as a single adjustment covariate in the BKMR models. Each BKMR model fit used 50,000 Markov Chain Monte Carlo iterations, and the trace plots of model parameters were visually examined to confirm model convergence. Results of the mixture analysis were summarized with the posterior inclusion probabilities (PIPs) and cross-section plots of the exposure response function. The association of the PFAS mixture with risk of GDM was examined by re-estimating the BKMR model with the PFAS analyte of interest forced into the mixture and used posterior samples to estimate the odds ratio (OR) and 95% credible intervals (95% CI) for GDM by 10% increments of each single PFAS, with all other PFAS set to the median.

Analyses were conducted using SAS version 9.4 and R version 4.1.0 with the bkmr R package for mixture models [63]. To account for multiple comparisons, p-values ($\alpha$=0.05) were false discovery rate (FDR) adjusted for the weighted unconditional logistic regression models.

## Results

Our sample included 128 participants at the baseline visit (CV1) in early pregnancy (41 cases, 87 controls) and 113 at CV2 in mid-pregnancy (29 cases, 84 controls). Participants were 32 ± 5 years of age and primarily Hispanic (39.1%) or Asian/Pacific Islander (25.0%), followed by White (21.9%), Black (7%), and 7% of individuals from other racial/ethnic groups. The majority were multiparous (55.9%). Participant characteristics of this sample were similar to the larger PETALS cohort [64] and characteristics for the GDM cases in PETALS (*n* = 310) and by timepoints of each clinic visit (*n* = 41 CV1, *n* = 29 CV2) are shown in Supplemental Table 1. Demographics for the sample and by GDM status are shown in Table 1.

**Table 1:** Participant Characteristics by GDM Cases and non-GDM Controls nested in the PETALS cohort: 2014–2017

	**All (*N* = 128)**	**GDM Cases (*N* = 41)**	**Controls (*N* = 87)**	*P*-value^*^
Age, years, mean ± SD	31.9 ± 4.9	32.4 ± 5.2	31.6 ± 4.8	0.42^a^
Race/ethnicity, n (%)				0.36^b^
Asian/Pacific Islander	32 (25.0%)	14 (34.2%)	18 (20.7%)	
Black	9 (7.0%)	2 (4.9%)	7 (8.0%)	
Hispanic	50 (39.1%)	16 (39.0%)	34 (39.1%)	
White	28 (21.9%)	8 (19.5%)	20 (23.0%)	
Other	9 (7.0%)	1 (2.4%)	8 (9.2%)	
Pre-pregnancy BMI, kg/m^2^, n (%)				0.06^b^
< 25.0 (Underweight/Normal)	44 (34.4%)	9 (22.0%)	35 (40.2%)	
25.0–29.9 (Overweight)	41 (32.0%)	13 (31.7%)	28 (32.2%)	
≥ 30.0 (Obese)	43 (33.6%)	19 (46.3%)	24 (27.6%)	
Nulliparity, n (%)	56 (44.1%)	14 (35.0%)	42 (48.3%)	0.16^b^
Early Pregnancy Blood Sample, gestational weeks, mean ± SD	13.8 ± 2.2	13.3 ± 2.1	14.0 ± 2.1	0.06^a^
Mid-Pregnancy Blood Sample, gestational weeks, mean ± SD	20.2 ± 2.2	19.5 ± 2.2	20.4 ± 2.2	0.07^a^

*BMI* Body mass index, *GDM* Gestational diabetes

^*^Obtained by ^a^Student's t test for continuous variables or ^b^Pearson's χ^2^ test for categorical variables

Seven of the fourteen measured PFAS analytes met our LOD inclusion criteria: PFHxS, PFOS, PFOA, PFNA, PFDA, PFUnDA, and PFPeA. Median PFAS concentrations were similar between the two timepoints. Distributions at each time point and the AUC are shown in Table 2. The paired concentrations of participants with samples at both clinic visits (*n* = 113) showed each PFAS to be moderately to highly correlated with one another (rho 0.53–0.94) and are shown in Supplemental Fig. 3. Within each separate timepoint, most PFAS were positively correlated with one another (rho 0.18 to 0.78), except for PFPeA (rho 0.09 to -0.16) (Supplemental Fig. 3). Significant differences in median concentrations by race/ethnicity groups were observed for all analytes except PFPeA, with highest medians observed in Asian/Pacific Islander and Black participants. Differences in medians were also identified by parity and pre-pregnancy BMI groups (Supplemental Table 2).

**Table 2:** Distributions for Pregnancy Serum PFAS Analytes in the PETALS cohort: 2014–2017

Analyte	% Detected^*^	**Median (IQR)**
**Perfluorodecanoic acid (PFDA), ng/mL**		
Early Pregnancy	87.5%	0.09 (0.04, 0.14)
Mid-Pregnancy	90.3%	0.07 (0.04, 0.13)
Area under the curve^a^	NA	13.24 (6.90, 23.51)
**Perfluorohexane-1-sulphonic acid (PFHxS), ng/mL**		
Early Pregnancy	100%	1.20 (0.91, 1.61)
Mid-Pregnancy	100%	1.23 (1.01, 1.68)
Area under the curve^a^	NA	222.55 (174.49, 295.24)
**Perfluorononanoic acid (PFNA), ng/mL**		
Early Pregnancy	100%	0.39 (0.28, 0.51)
Mid-Pregnancy	100%	0.35 (0.26, 0.47)
Area under the curve^a^	NA	70.13 (50.68, 89.37)
**Perfluorooctanesulfonic acid (PFOS), ng/mL**		
Early Pregnancy	100%	2.46 (1.67, 3.40)
Mid-Pregnancy	100%	2.42 (1.69, 3.30)
Area under the curve^a^	NA	438.11 (316.51, 634.87)
**Perfluorooctanoic acid (PFOA), ng/mL**		
Early Pregnancy	100%	0.71 (0.46, 1.07)
Mid-Pregnancy	100%	0.69 (0.47, 0.97)
Area under the curve^a^	NA	130.72 (87.47, 188.41)
**Perfluoroundecanoic acid (PFUnDA), ng/mL**		
Early Pregnancy 1	93.8%	0.10 (0.05, 0.19)
Mid-Pregnancy 2	93.8%	0.10 (0.05, 0.17)
Area under the curve^a^	NA	14.87 (9.22, 33.90)
**Perfluoro-n-pentanoic acid (PFPeA), ng/mL**		
Early Pregnancy 1	100%	0.28 (0.20, 0.42)
Mid-Pregnancy 2	99.1%	0.29 (0.19, 0.43)
Area under the curve^a^	NA	53.40 (37.74, 76.75)

*NA* Not applicable

^*^Limit of detection (LOD) for PFHxS, PFDA, PFOA, PFUnDA, PFOS = 0.02 ng/mL; PFPeA = 0.0224 ng/mL; PFNA = 0.032 ng/mL

Early pregnancy samples collected at 13.9 $\pm$ 2.1 weeks of gestation and mid-pregnancy samples collected at 20.1 $\pm$ 2.2 weeks of gestation

^a^Calculated (in ng/mL × day) using the formula: $M1\times D1+\frac{(M1+M2)\times (D2-D1)}{2}+M2\times (197-D2)$, where M1 and M2 were concentrations of PFAS at each time point, D1 and D2 were days of gestation at the two time points, respectively, and 197 was the maximum D2 within this sample

In multivariable models shown in Table 3, early pregnancy concentrations, mid-pregnancy concentrations, and the AUC of PFDA, PFNA and PFOA were individually significantly associated with higher GDM risk. Specifically, for early pregnancy the following associations with GDM risk were found: PFDA (OR: 1.23 [95% CI 1.09, 1.38]), PFNA (OR:1.40 [95% CI 1.24, 1.58]), and PFOA (OR:1.15 [95% CI 1.04, 1.27]) per IQR. For mid-pregnancy, we observed the following associations: PFDA (OR:1.28 [95% CI 1.15, 1.43]), PFNA (OR:1.16 [95% CI 1.05, 1.28]), and PFOA (OR:1.20 [95% CI 1.09, 1.33]) per IQR. Finally, for the AUC the associations of each of these PFAS and GDM risk were as follows: PFDA (OR:1.23 [95% CI 1.09, 1.38]), PFNA (OR:1.21 [95% CI 1.07, 1.37]), and PFOA (OR:1.19 [95% CI 1.09, 1.31]) per IQR (Table 3). Early pregnancy concentrations of PFOS were not associated with GDM risk, however, mid-pregnancy concentrations and the AUC for PFOS were individually associated with higher GDM risk (OR:1.41 [95% CI 1.17, 1.71] and OR: 1.33 [95% CI 1.06, 1.58], per IQR, respectively). For PFUnDA, early pregnancy levels were associated with lower GDM risk (OR: 0.79 per IQR [95% CI 0.64, 0.98]), whereas mid-pregnancy levels were associated with higher GDM risk (OR:1.49 per IQR [95% CI 1.18, 1.89]). For PFHxS, concentrations were associated with decreased risk of GDM in early pregnancy (OR:0.48 [95% CI 0.38, 0.60]), mid-pregnancy (OR: 0.48 [95% CI 0.37, 0.63]) and with the AUC (OR: 0.49 [95% CI 0.38, 0.63]) per IQR. PFPeA was not associated with GDM risk at either timepoint or with the AUC (Table 3).

**Table 3:**
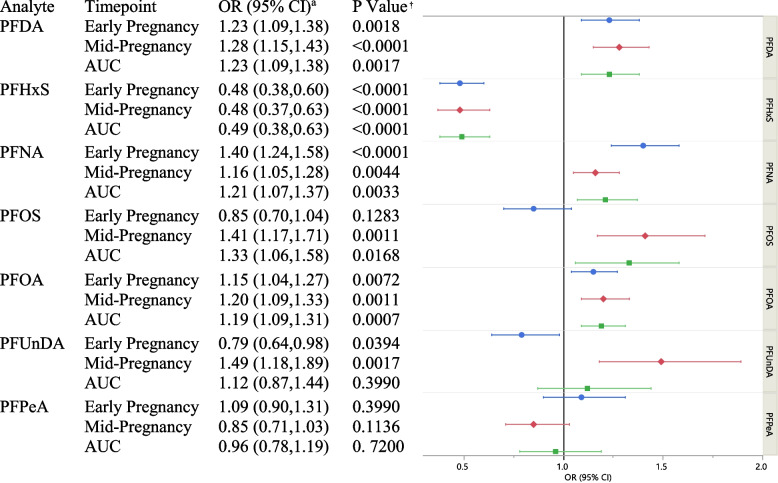
Adjusted Odds Ratio (95% CI) for the Association of Pregnancy Serum PFAS with Gestational Diabetes: a Nested Case–Control Study in the PETALS Cohort: 2014–2017

Participants completed Clinic Visit 1 in early pregnancy on average at 13.9 ± 2.2 weeks of gestation and Clinic Visit 2 in mid pregnancy on average at 20.1 ± 2.2 weeks of gestation

The average time between clinic visits was 6.4 (SD: 0.95) weeks. OR (95% CI) is per log IQR (interquartile range) increment

^†^*P*-value adjusted for false discovery rate (FDR)

^a^Models adjusted for age, pre-pregnancy BMI, parity, and race/ethnicity

**Fig. 2:**
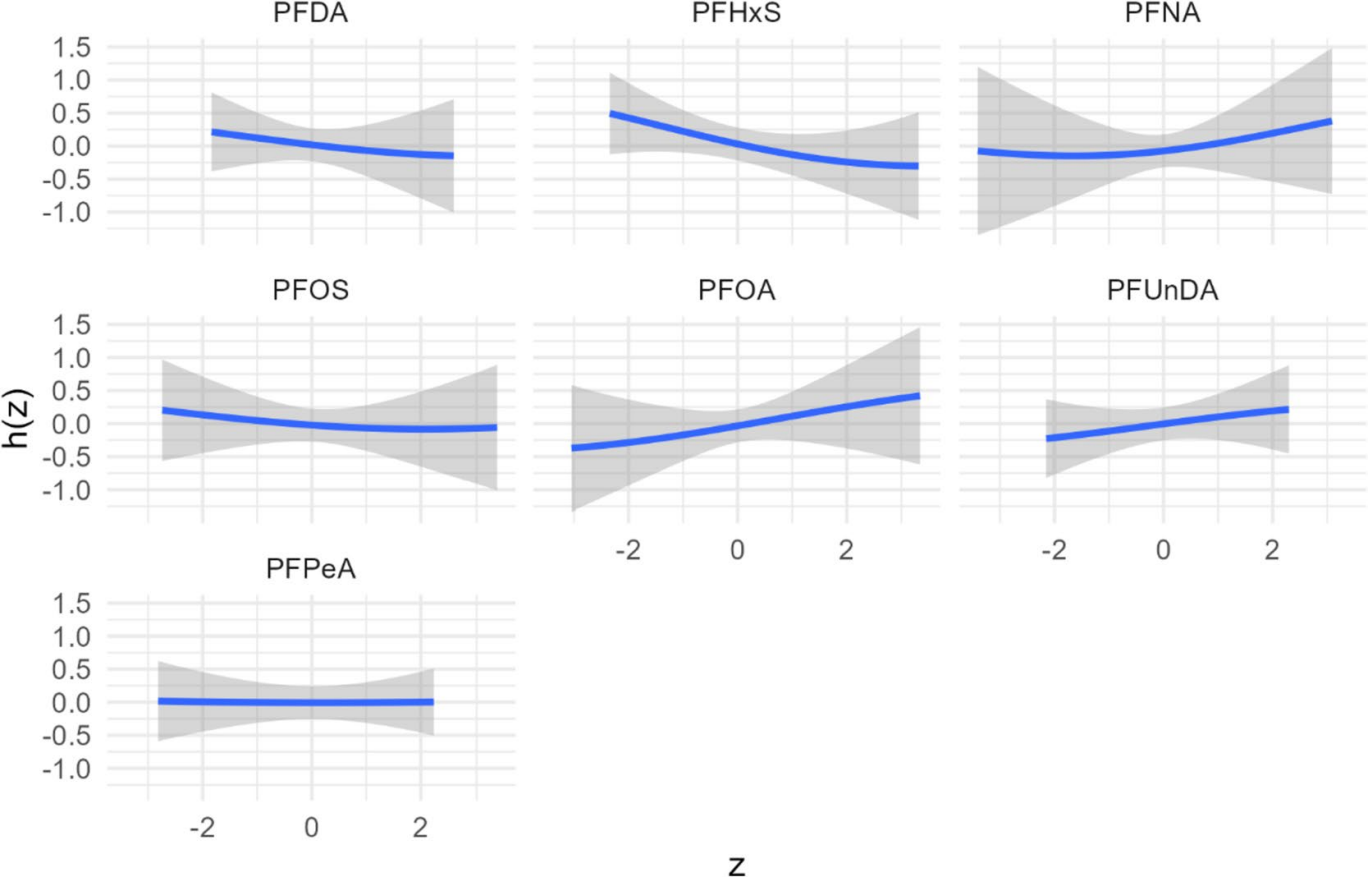
Exposure response function for each PFAS from Bayesian kernel machine regression models with the Area Under the Curve (AUC) between the two clinic visits

**Fig. 3:**
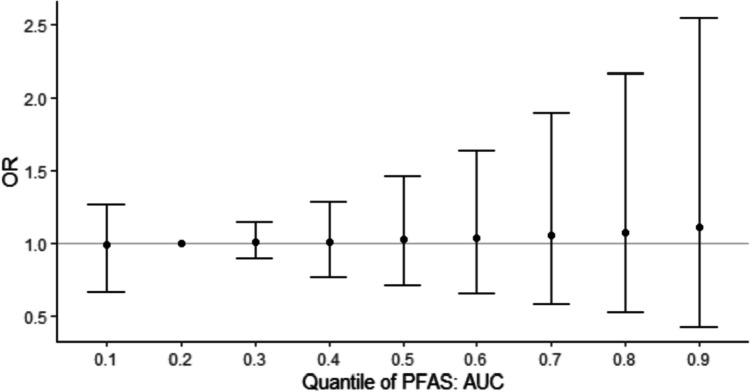
Overall PFAS Mixture Effect with Odds of GDM, Area Under the Curve

In mixture analyses, PFHxS had the largest contribution to the mixture (Supplemental Table 3), although PIPs for each PFAS were moderate (Early Pregnancy: 0.31–0.61; Mid-Pregnancy: 0.34–0.51). In the cross-section plots of the exposure response function for each PFAS, PFNA, PFOA and PFUnDA showed suggestive positive associations with GDM risk, while PFHxS suggested an inverse association with GDM risk (Fig. 2 (AUC) and Supplemental Figs. 4–5). No significant associations were found at any timepoint for the overall mixture effect via summary ORs (Fig. 3 (AUC) and Supplemental Figs. 6–7).

## Discussion

This nested case–control study within the racially and ethnically diverse PETALS cohort found consistent evidence that prenatal exposure to PFAS, notably PFDA, PFNA, and PFOA, during early and mid-pregnancy was significantly associated with higher risk of GDM. For PFOS and PFUnDA, increased risk of GDM was observed only for exposure during mid-pregnancy. In contrast, PFHxS was associated with decreased risk of GDM during early and mid-pregnancy. Mixture results suggested similar findings, but overall mixture models were not statistically significant.

The current literature of PFAS exposure during gestation and risk of GDM has primarily been assessed using PFAS levels collected at only one timepoint in pregnancy. Although PFAS have long half-lives, analyte levels overall have been shown to vary across pregnancy in the same individual [49], and critical windows of exposure to PFAS may occur during gestation. It is also possible that maternal metabolic shifts occur including different rates of transplacental PFAS transfer to the fetus and blood volume expansion or mobilization of stored PFAS across pregnancy [65, 66] which may explain inverse associations or the possibility of reverse causation for samples collected late in pregnancy [67]. In our study, results using early pregnancy samples compared to mid-pregnancy samples were relatively consistent, although different conclusions were made for PFUnDA and PFOS across timepoints which may indicate less evidence for exposure to these analytes with risk of GDM. It is also possible that the physiological changes occurring during mid-pregnancy, such as increased insulin resistance and hormonal shifts in mid to late pregnancy may interact with PFOS and PFUnDA exposure differently compared to earlier in pregnancy [68]. An example includes Human Placental Lactogen (hPL) which is produced by the placenta and helps regulate maternal metabolism and fetal growth which steadily increases during the second trimester and works to decrease the mother's sensitivity to insulin, making more glucose available for the growing fetus [69].

Higher levels of PFAS have been associated with type 2 diabetes [70, 71], increased glucose and insulin levels in pregnancy [17] and may play an important role in GDM development [18]. During pregnancy, the body becomes more resistant to insulin due to hormonal changes and PFAS exposure may exacerbates this insulin resistance, thereby increasing the risk of GDM [68]. Proposed mechanisms of PFAS influences on glucose levels observed in pregnancy include inflammation and oxidative stress which can impair insulin signaling and glucose metabolism, alterations in fatty acid and adipose tissue disfunction including changes in adipokine secretion and adipocyte differentiation, which is associated with insulin resistance, and activation of peroxisome proliferator-activated receptors (PPARs) based on toxicological evidence [72, 73]. However, previous epidemiological studies on the association between PFAS exposure and GDM risk are inconclusive due to suggesting differential risks of certain analytes with GDM risk.

In studies conducted within the United States examining the association between PFAS exposure during gestation and GDM risk, PFAS were assessed either before pregnancy [74] or in the first trimester [46, 75] and included populations of primarily non-Hispanic White participants, except for Rahman et al. which included a diverse study population [75]. Our findings that PFOA and PFNA were associated with greater risk of GDM is consistent with results from two previous US cohorts [74, 75] though a third reported null associations with PFOA but observed PFOS to be a primary driver within BKMR models for increased continuous glucose levels [46]. PFOS and PFNA had significant positive associations with blood glucose levels among Asian participants with the largest effect estimates compared to other racial groups [46]. The inverse association found with PFHxS has not been replicated in other studies [41, 43–45, 47]. However, inverse associations have been reported with other cardiometabolic conditions in pregnancy from prenatal PFHxS exposure [76]. This inverse association with blood pressure in pregnancy was thought to be a chance finding from the authors due to not having a biological mechanism known that would explain this relationship [76]. It is possible that the inverse associations in this current study may also be due to chance even after FDR adjusting the p-values. Other hypotheses include random variation in PFHxS due to the small sample size creating potential noise in the data, a complex interaction with other PFAS compounds in that PFHxS may interact with other compounds in ways that are not yet fully understood which has potentially led to this unexpected association, as well as potential effect modification by a third variable (i.e., race/ethnicity, pre-pregnancy BMI, or maternal age), which we were interested in assessing but were unable to produce reliable estimates due to the small sample sizes within each strata.

Studies conducted in Chinese populations produced results that have been inconclusive. Wang et al. found that early pregnancy levels of PFOA and PFOS were not associated with GDM risk [40], however, PFOA was significantly associated with fasting insulin and homeostasis model assessment of insulin resistance [40]. Xu et al. found PFBS and PFDoA to be positively associated with GDM risk, but null for PFAS that overlapped in this study [42]. Liu et al. used summed values based on structural characteristics of PFAS and found total perfluoroalkyl carboxylates (including PFOA, PFNA, PFDA, PFUnDA among others) to be positively associated with GDM risk [38], which is consistent with analytes showing higher risk of GDM within our results. Yu et al. conducted a BKMR model and found that the PFAS mixture exposure was positively associated with GDM incidence and PFOS, PFNA and PFHpA were main contributors to the mixture [43]. This was inconsistent with our findings as PFHxS was a primary contributor although PFNA was the secondary contributor for both timepoints and the AUC. Our mixture analysis through BKMR was also not statistically significant and we hypothesize that the small sample size was a hinderance and that the complex inverse and positive associations across analytes may have made the mixture difficult to disentangle.

Prominent strengths of this study include the longitudinal design, diverse study population from an integrated health care system with universal screening for GDM, the availability of pregnancy levels of glycemia for rigorous and standardized assessment of GDM and multiple time points for PFAS concentration assessment within pregnancy which held temporality due to being collected prior to GDM ascertainment. This study also incorporated mixture methods using BKMR which has been underutilized and identified as a gap within the current literature [71]. Mixture approaches are important when assessing these associations due to the concurrent and correlated nature of the exposures.

Limitations are also present. The sample size is relatively small, but the original 2–1 matching design increased statistical power for the individual models and all results were FDR-adjusted to reduce the risk of chance findings. The mixture models suggested similar conclusions to the individual models but did not reach statistical significance, likely due to not being well powered for BKMR models. Additionally, low detection frequencies (< 50% of samples) for seven of the fourteen PFAS inhibited inclusion. Lastly, true overall exposure to PFAS across early to mid-pregnancy was not obtained and instead used the AUC as a proxy measurement. However, AUC results were consistent to early and mid-pregnancy results.

## Conclusions

This prospective nested case–control study observed that higher serum levels of PFDA, PFNA, and PFOA during early and mid-pregnancy were consistently associated with higher GDM risk in an ethnically diverse population. It is important to corroborate results in larger sample sizes and to potentially advise individuals of reproductive age to avoid known sources of PFAS.

### Supplementary Information

**Additional file 1: Supplemental Figure 1.** Directed acyclic graph (DAG) Reflecting the Total Effect of Gestational PFAS Exposure on GDM. **Supplemental Figure 2.** Area Under the Curve (AUC) Equation. **Supplemental Figure 3.** Spearman Correlations of PFAS Analytes by Timepoint. **Supplemental Table 1.** Demographics of GDM Cases in the PETALS Cohort and those Included in Current Study. **Supplemental Table 2.** Median PFAS Analyte Concentrations (ng/mL) by Participant Characteristics. **Supplemental Table 3.** Posterior Inclusion Probabilities of each PFAS analyte in Bayesian kernel machine regression models. **Supplemental Figure 4.** Exposure response function for each PFAS from Bayesian kernel machine regression model in early pregnancy (Clinic Visit 1). **Supplemental Figure 5.** Exposure response function for each PFAS from Bayesian kernel machine regression model among in mid-pregnancy (Clinic Visit 2). **Supplemental Figure 6.** Overall PFAS Mixture Effect with Odds of GDM, Clinic Visit 1. **Supplemental Figure 7.** Overall PFAS Mixture Effect with Odds of GDM, Clinic Visit 2.

## Data Availability

Extracted data are available within the publication and its supplementary files. A de-identified analytic dataset with code used in this study can be shared with qualified researchers subject to approval by the Kaiser Foundation Research Institute Human Subjects Committee and by the Human Subjects Committee at the institutions requesting the data and a signed data sharing agreement. Please send all requests to the corresponding author of this article. Data will be available to requesters from 1 year after the date of publication of this article.

## Abbreviations

BKMR: Bayesian kernel machine regression
CV1: Clinic Visit 1
CV2: Clinic Visit 2
EDCs: Endocrine-disrupting chemicals
FDR: False discovery rate
GDM: Gestational diabetes mellitus
IQR: Interquartile range
KPNC: Kaiser Permanente Northern California
LOD: Limit of detection
OR: Odds ratio
OGTT: Oral glucose tolerance test
NETFOSAA: N-ethyl perfluorooctane sulfonamido acetic acid
NMFOSAA: N-methyl perfluorooctane sulfonamido acetic acid
PFAS: Per- and polyfluoroalkyl substances
PFOA: Perfluorooctanoic acid
PFOS: Perfluorooctanesulfonic acid
PFHxS: Perfluorohexanesulfonic acid
PFDA: Perfluorodecanoic acid
PFNA: Perfluorononanoic acid
PFUnDA: Perfluoroundecanoic acid
PFPeA: Perfluoro-n-pentanoic acid
PFBS: Perfluorobutanesulfonic acid
PFHPA: Perfluoroheptanoic acid
PFDODA: Perfluorododecanoic
PFOSA: Perfluorooctanesulfonamide
PFHxA: Perfluorohexanoic acid
PIPs: Posterior inclusion probabilities
CI: 95% Confidence Interval
