# Supplementary material for: PFAS concentrations in early and mid-pregnancy and risk of gestational diabetes mellitus in a nested case-control study within the ethnically and racially diverse PETALS cohort
Source: BMC Pregnancy Childbirth. 2023 Sep 13;23:657. doi: 10.1186/s12884-023-05953-3 (PMC10500777; doi:10.1186/s12884-023-05953-3)
Supplement: Supplementary file 1 — Additional file 1: Supplemental Figure 1. Directed acyclic graph (DAG) Reflecting the Total Effect of Gestational PFAS Exposure on GDM. Supplemental Figure 2. Area Under the Curve (AUC) Equation. Supplemental Figure 3. Spearman Correlations of PFAS Analytes by Timepoint. Supplemental Table 1. Demographics of GDM Cases in the PETALS Cohort and those Included in Current Study. Supplemental Table 2. Median PFAS Analyte Concentrations (ng/mL) by Participant Characteristics. Supplemental Table 3. Posterior Inclusion Probabilities of each PFAS analyte in Bayesian kernel machine regression models. Supplemental Figure 4. Exposure response function for each PFAS from Bayesian kernel machine regression model in early pregnancy (Clinic Visit 1). Supplemental Figure 5. Exposure response function for each PFAS from Bayesian kernel machine regression model among in mid-pregnancy (Clinic Visit 2). Supplemental Figure 6. Overall PFAS Mixture Effect with Odds of GDM, Clinic Visit 1. Supplemental Figure 7. Overall PFAS Mixture Effect with Odds of GDM, Clinic Visit 2. [file 12884_2023_5953_MOESM1_ESM.docx]

**Supplemental Material**

Supplemental Methods

*PFAS Concentration Analysis*

The detailed PFAS analysis is as described. Serum samples (0.25 mL) were aliquoted into 15 mL polypropylene (PP) tubes and spiked with 5 ng of 13C-labeled internal standard (IS) mixture and 0.7 mL of 1% ammonium formate (w/v) in methanol (MeOH). The mixture was centrifuged for 5 min at 5000 rpm and the supernatant was collected and passed through Hybrid-SPE cartridge (Phospholipid, 30mg, 1cc, Sigma-Aldrich, St. Louis, MO, USA). The cartridges were conditioned with 1 mL of MeOH containing 1% ammonium formate (w/v). The samples were eluted through the cartridge and collected in a PP tube for LC-MS/MS analysis. Target chemicals were analyzed using an ABSCIEXTM 5500 electrospray tandem quadrupole mass spectrometer (ESI-MS/MS: SCIEX, Framingham, MA, USA), interfaced with a Nexera X2 LC-30AD series HPLC (Shimadzu, Kyoto, Japan). The analytes were separated using an UPLC BEH C18 column (2.1 x 50 mm, 1.7 µm, Waters, Milford, MA, USA). MeOH and 0.1% w/v ammonium acetate in water were used as mobile phases. Target analytes were determined by selected reaction monitoring (SRM) in negative ionization mode. Further details of mass spectrometric conditions and LC parameters are described in Honda et al. The target analytes were quantified by isotopic dilution method and a 12-point calibration (at concentrations ranging from 0.02 to 100 ng/mL) with the regression coefficient of ≥ 0.999 was used. A pure solvent (MeOH) and a mid-point calibration standard (5 ng/mL) were injected after every 10 samples to check for carry over of target chemicals and instrumental drift in sensitivity. Several procedural blanks were analyzed to monitor for contamination that can arise from reagents and materials used in sample preparation steps. For each batch of 100 samples, five replicates of procedural blanks and QC spiked samples (water spiked with native standards at 5 ng for all analytes and IS) were processed. Also, two replicates of Standard Reference Material (SRM1957 and SRM1958, NIST, Gaithersburg, MD, USA; IS spiked) containing certified values for PFHxS, PFOS, PFHpA, PFOA, PFNA, PFDA, and PFUnDA for SRM1957 and PFHxS, PFOS, PFOA, and PFNA for SRM1958 were processed. Trace levels of PFPeA (0.001 – 0.009 ng/mL), PFNA (0.001 – 0.008 ng/mL), PFDA (0.001 – 0.005 ng/mL), PFUnDA (0.001 – 0.008 ng/mL), and N-MeFOSAA (0.001 – 0.009 ng/mL) were found in procedural blanks, and the concentrations of these chemicals in samples were subtracted from blank values. QC spiked recoveries, SRM1957, and SRM1958 recoveries were 78.1-130.3% (RSD: ±4.2-11.3%), 60.5-103.2% (RSD: ±4.7-14.3%), and 85.6-110.4% (RSD: ±2.8-6.1%) respectively. The limit of detection of target analytes ranged from 0.02 to 0.032 ng/mL.

**Supplemental Figure 1. Directed acyclic graph (DAG) Reflecting the Total Effect of Gestational PFAS Exposure on GDM**


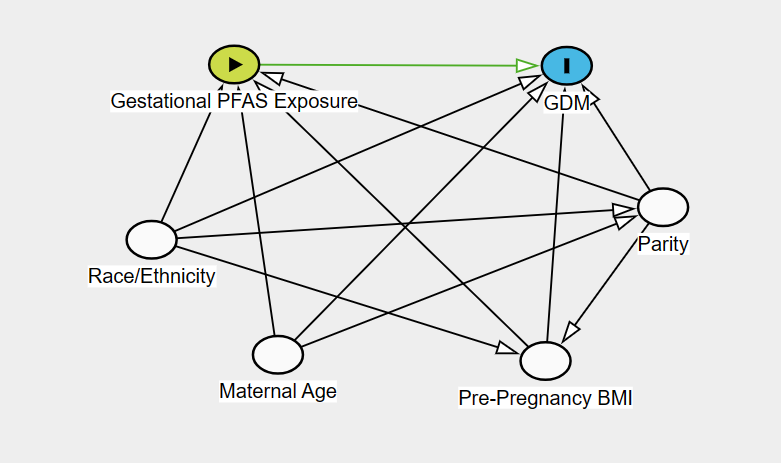


**Supplemental Figure 2. Area Under the Curve (AUC) Equation**

$$M1\times D1+\frac{(M1+M2)\times(D2-D1)}{2}+M2\times(197-D2)$$

Calculated in ng/mL × day where M1 and M2 were concentrations of PFAS analytes at each time point, D1 and D2 were days of gestation at the two time points, respectively, and 197 was the maximum D2 based on the distribution in the entire PETALS cohort.

**Supplemental Figure 3. Spearman Correlations of PFAS Analytes by Timepoint**


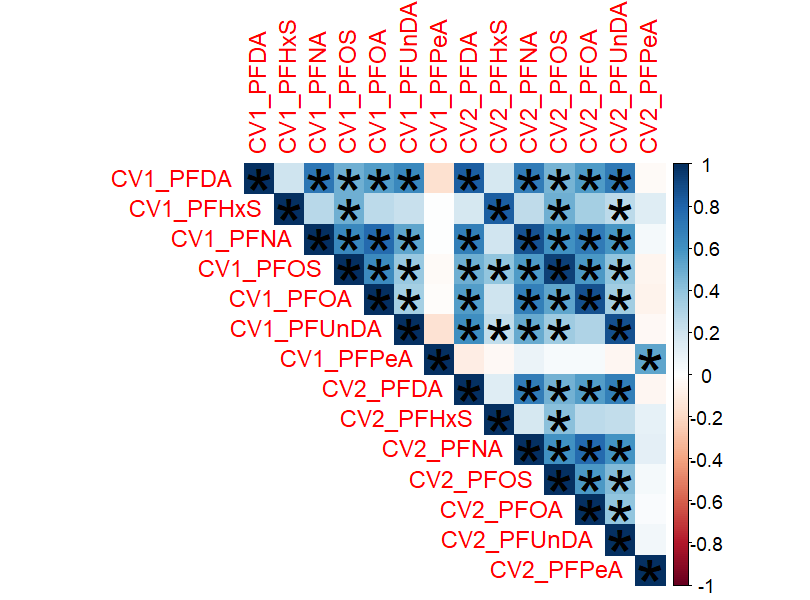


Note:CV1=Clinic Visit 1 CV2= Clinic Visit 2; *=p<0.05

**Supplemental Table 1. Demographics of GDM Cases in the PETALS Cohort and those Included in Current Study**

|  | **All 310 GDM Cases in PETALS** | **41 GDM Cases for CV1 analysis in current study** | **29 GDM Cases for CV2 analysis in current study** |
| --- | --- | --- | --- |
| **BMI**  Normal  Overweight  Obese | 71 (22.9%)  86 (27.7%)  153 (49.4%) | 9 (22.0%)  13 (31.7%)  19 (46.3%) | 6 (20.7%)  10 (34.5%)  13 (44.8%) |
| **Age** | 31.7 ± 4.9 | 32.7 ±5.1 | 32.3±5.6 |
| **Race/Ethnicity** Asian/Pacific Islander  Black  Hispanic  Other White | 120 (38.7%)  14 (4.5%)  120 (38.7%)  10 (3.3%) 46 (14.8%) | 14 (34.1%)  2 (5.0%)  16 (39.0%) 1 (2.4%) 8 (19.5%) | 8 (27.6%)  0 (0%)  12 (41.4%)  1 (3.4%)  8 (27.6%) |
| **Parity**  Nulliparous | 130 (41.9%) | 14 (34.1%) | 10 (34.4%) |

CV1= Clinic Visit 1; CV2 Clinic Visit 2

**Supplemental Table 2. Median PFAS Analyte Concentrations (ng/mL) by Participant Characteristics**

|  | **PFDA** | **PFHxS** | **PFNA** | **PFOA** | **PFOS** | **PFUnDA** | **PFPeA** |
| --- | --- | --- | --- | --- | --- | --- | --- |
| **GDM Status^b^** |  |  |  |  |  |  |  |
| Early Pregnancy |  |  |  |  |  |  |  |
| GDM Case | 0.09 | 1.02* | 0.39 | 0.73 | 2.40 | 0.10 | 0.28 |
| Control | 0.08 | 1.26* | 0.39 | 0.70 | 2.51 | 0.08 | 0.29 |
| Mid-Pregnancy |  |  |  |  |  |  |  |
| GDM Case | 0.06 | 1.08* | 0.35 | 0.70 | 2.30 | 0.09 | 0.29 |
| Control | 0.07 | 1.26* | 0.37 | 0.68 | 2.42 | 0.10 | 0.30 |
| **Race/ethnicity^a^** |  |  |  |  |  |  |  |
| Early Pregnancy |  |  |  |  |  |  |  |
| Asian/Pacific Islander | 0.14** | 1.20* | 0.52* | 0.79 | 2.78* | 0.20** | 0.30 |
| Black | 0.08** | 1.28* | 0.44* | 0.89 | 3.07* | 0.17** | 0.24 |
| Hispanic | 0.06** | 1.06* | 0.35* | 0.59 | 2.06* | 0.07** | 0.30 |
| White | 0.07** | 1.52* | 0.34* | 0.80 | 2.73* | 0.06** | 0.26 |
| Other | 0.07** | 1.26* | 0.34* | 1.01 | 2.45* | 0.11** | 0.37 |
| Mid-Pregnancy |  |  |  |  |  |  |  |
| Asian/Pacific Islander | 0.13* | 1.17 | 0.48** | 0.75* | 2.74* | 0.20** | 0.27 |
| Black | 0.08* | 1.17 | 0.36** | 0.83* | 2.90* | 0.10** | 0.34 |
| Hispanic | 0.06* | 1.13 | 0.29** | 0.55* | 2.02* | 0.07** | 0.32 |
| White | 0.06* | 1.59 | 0.33** | 0.78* | 2.52* | 0.08** | 0.26 |
| Other | 0.07* | 1.51 | 0.34** | 0.87* | 2.44* | 0.12** | 0.36 |
| **Pre-pregnancy BMI, kg/m^2 a^** |  |  |  |  |  |  |  |
| Early Pregnancy |  |  |  |  |  |  |  |
| <25.0 | 0.10* | 1.46* | 0.41 | 0.79 | 2.51 | 0.17** | 0.27 |
| 25.0-29.9 | 0.09* | 1.19* | 0.39 | 0.72 | 2.48 | 0.10** | 0.28 |
| ≥30.0 | 0.06* | 1.08* | 0.35 | 0.65 | 2.21 | 0.06** | 0.30 |
| Mid-Pregnancy |  |  |  |  |  |  |  |
| <25.0 | 0.09 | 1.58* | 0.37 | 0.71 | 2.59 | 0.15** | 0.28 |
| 25.0-29.9 | 0.07 | 1.20* | 0.38 | 0.61 | 2.41 | 0.08** | 0.29 |
| ≥30.0 | 0.04 | 1.06* | 0.33 | 0.70 | 2.24 | 0.07** | 0.29 |
| **Parity^b^** |  |  |  |  |  |  |  |
| Early Pregnancy |  |  |  |  |  |  |  |
| Nulliparous | 0.09 | 1.25 | 0.44* | 0.93** | 2.75* | 0.11 | 0.29 |
| Parous | 0.08 | 1.17 | 0.35* | 0.59** | 2.31* | 0.08 | 0.28 |
| Mid-Pregnancy |  |  |  |  |  |  |  |
| Nulliparous | 0.09 | 1.24 | 0.40* | 0.91** | 2.81* | 0.12 | 0.30 |
| Parous | 0.06 | 1.18 | 0.32* | 0.59** | 2.14* | 0.08 | 0.27 |
| PFDA: Perfluorodecanoic acid, PFHxS: Perfluorohexane-1-sulphonic acid, PFNA: Perfluorononanoic acid, PFOS: Perfluorooctanesulfonic acid, PFOA: Perfluorooctanoic acid, PFUnDA: Perfluroundecanoic acid, PFPeA: Perfluoro-n-pentanoic acid  ^a^ P-value from Kruskal Wallis test ^b^ P-value from Wilcoxon Rank Sum test  *Significant at p < 0.05; **Significant at p < 0.001 | | | | | | | |

**Supplemental Table 3.** Posterior Inclusion Probabilities of each PFAS analyte in Bayesian kernel machine regression models

|  | **Clinic Visit 1** | **Clinic Visit 2** | **AUC** |
| --- | --- | --- | --- |
| PFDA | 0.40 | 0.34 | 0.39 |
| PFHxS | 0.61 | 0.51 | 0.59 |
| PFNA | 0.47 | 0.48 | 0.51 |
| PFOS | 0.36 | 0.36 | 0.39 |
| PFOA | 0.43 | 0.37 | 0.45 |
| PFUnDA | 0.40 | 0.48 | 0.43 |
| PFPeA | 0.31 | 0.30 | 0.35 |

AUC: area under the curve across the two clinic visits

**Supplemental Figure 4.** Exposure response function for each PFAS from Bayesian kernel machine regression model in early pregnancy (Clinic Visit 1).


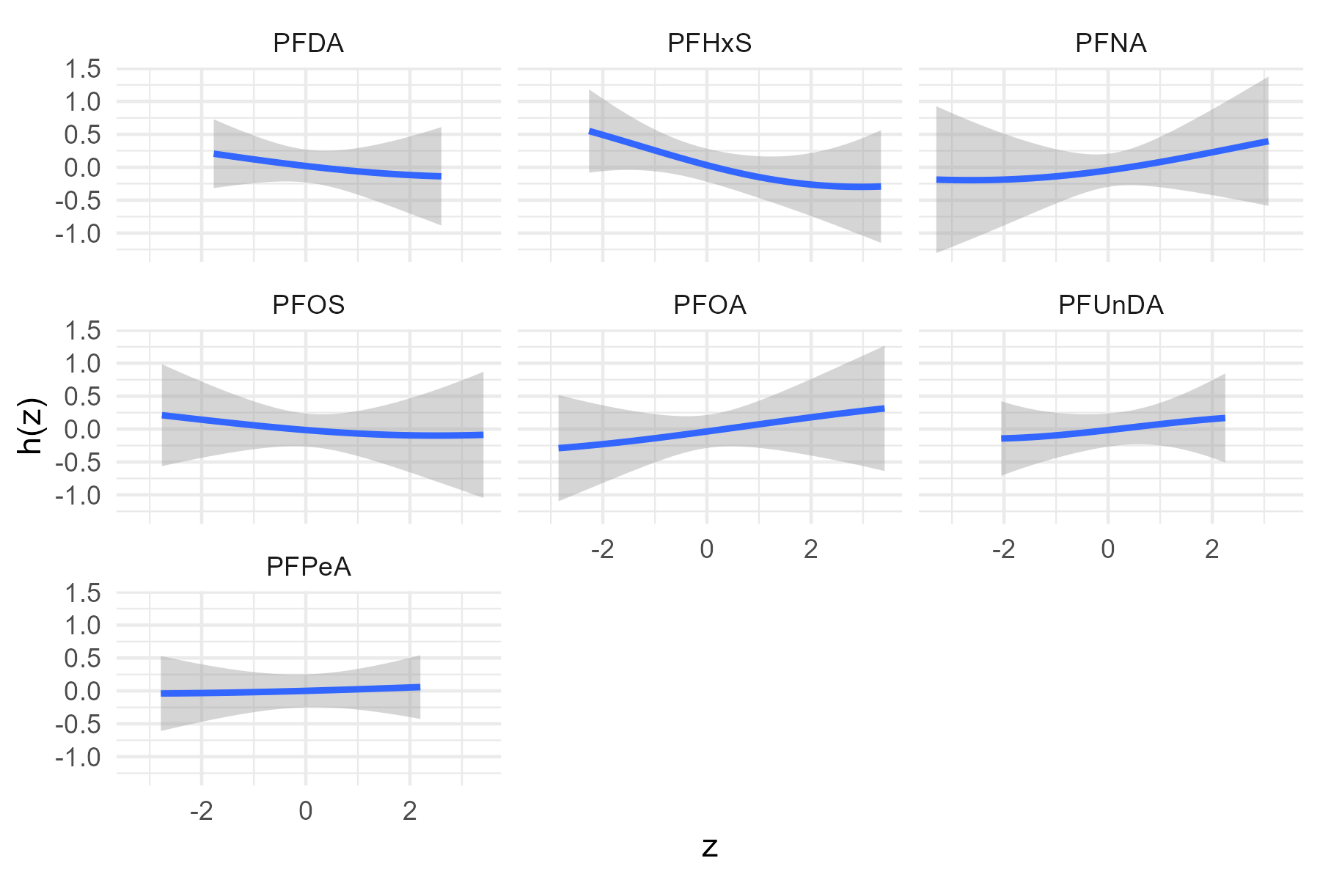


**Supplemental Figure 5**. Exposure response function for each PFAS from Bayesian kernel machine regression model among in mid-pregnancy (Clinic Visit 2)


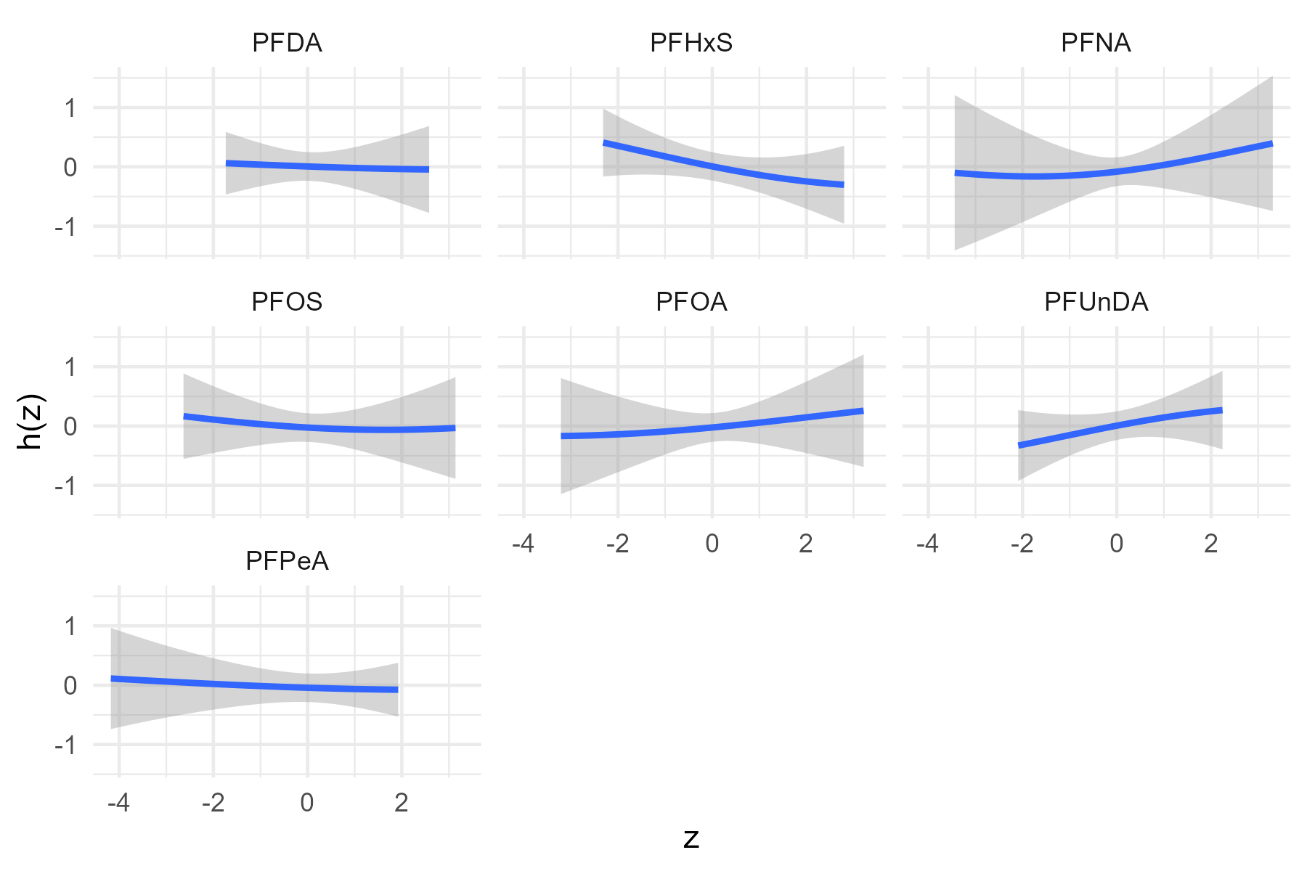


**Supplemental Figure 6.** Overall PFAS Mixture Effect with Odds of GDM, Clinic Visit 1


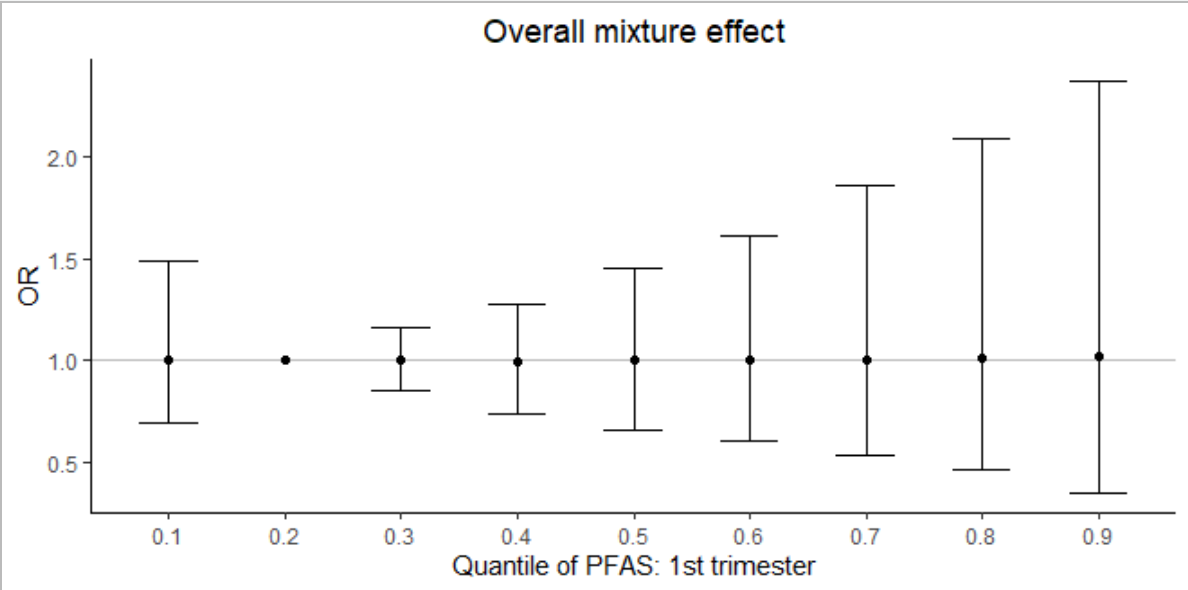


Quantile of PFAS: Early Pregnancy (CV1)

**Supplemental Figure 7.** Overall PFAS Mixture Effect with Odds of GDM, Clinic Visit 2


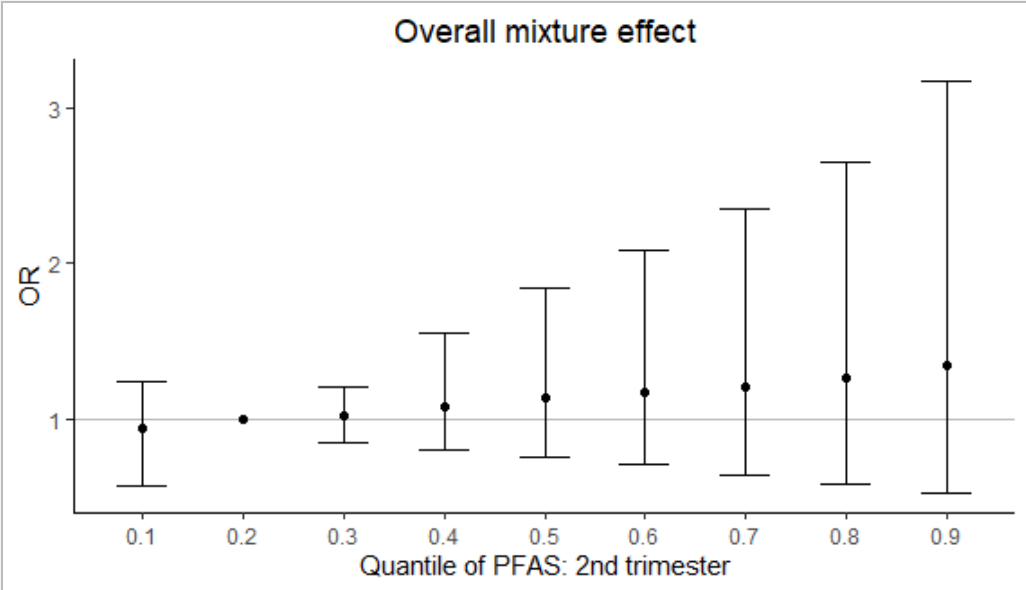


Quantile of PFAS: Mid-Pregnancy (CV2)
